# Dental Periodical Literature of Dentistry

**Published:** 1859-10

**Authors:** 


### ARTICLE V.

### *Dental Periodical Literature of Dentistry.*

No subject is more interesting to the observer of the progress of dentistry than the state of its literature, and more particularly, its periodical literature. It is difficult to say whether dentists should esteem it or not. If we look at its recent meagre beginnings and compare them with the present flood, we shall as far as numbers go, have much to congratulate ourselves upon; but if we reflect upon the quality of the material now offered, and the purpose for the accomplishment of which these dental pamphlets are projected, we shall rather regret their existence. Certainly outside and uninterested spectators, as the writer professes to be, can never be induced to respect the multitude of trashy performance which are spread broadcast over the country in weekly, monthly, and quarterly instalments, for no other object, as we have been able to discover, than to introduce some dealer of professional wares to professional notice. The inventor of a peculiar grindstone desires to present his machine to the "profession" in the most advantageous manner; the thing is easily done. One or two individuals are asked to furnish the, by no means edifying, contents of their scrap-books for publication. The distinguished writers cheerfuly respond to the complimentary proposition and hasten to become immortal in the pages of some equally immortal periodical which is sent with or without an annual subscription to every dentist in and out of the country. *A few* examples caricatured, from really eminent writers, furnish the frame work of these "scraps." "Mr." or "Mrs. A. B. or C. called at my office after having sought relief from several physicians and dentists, to have a left upper second molar extracted. I accomplished the operation with ease, by means of a forcep of my own invention, and on the third day the patient was comparatively well considering the severity of the operation." Of course we give no more than a bare outline of one of these reports. The verbiage and non-essential part swells this important communication into an "article" of from three to ten pages. Then we have notes from the workshop. We are informed that it is well to communicate special details of practice. Dr. or Prof. R. S. T. U. or W. imparts the important circumstance that borax is a good thing. We are edified with the information that plates sometimes warp and that it is very "hard" to get one to fit. Half a dozen, maybe more but sometimes less, of these efforts of genius fill up a "No." of some periodical speculation. The advertisements are really valuable, for they tell you something of things wanted; the literary or scientific matters, if it may be called such, reduces the advertising to the level of patent medicine almanacs and poetical sewing machine circulars.

Now we do not wish it to be understood that we object to the little tricks of trade. That were truly childish. They belong to humanity. Nor do we question the right of members of the profession to send post-paid pamphlets all over the world upon their own responsibility.

But we do object to the barefaced assumption of these men that they are the "profession;" we deny it. They do not form the camp. They are the "*sutlers*." We would appeal to the men of education and dignity, to stand forward and renew their efforts with the same zeal which actuated them in the beginning. Let them destroy the identity which exists in the popular mind between the "sutlers" and the soldiers. Let them not retire before the hawkers and criers. They should rather vindicate themselves and the profession which they have created, and which requires further care. It is true all have not deserted; two or three of the greatest names are still continually before us, still we want more of the veteran force.

With a few notable exceptions all that has been done for dentistry was done by the men who created it. Fifteen or twenty years has surely not made old and unqualified men of those who were then youthful. Why then should they supinely retire upon their thousands and millions, intimating that they have nothing to be thankful for. They should *work* for the honor of the profession which they took pride in. The contempt of the future otherwise awaits them. The earnest laborious sacrifices of the old and experienced should stimulate and dignify the efforts of their successors. We are thankful for all that has been done; for the real results of elevated motive and for the fruits which have been recently gathered in the shape of positive scientific contributions. We deprecate and disavow the noisy babble of busy pretenders.

The "profession" at present cannot furnish contributions of the proper kind to the immense number of periodicals which are published. We are sorry to admit it, but the truth must be told. Out of the four or five thousand dentists in this country a sufficient number might be, indeed have lately been, found to furnish one or two periodicals with the proper material. We are convinced of this from an intimate acquaintance with the facts, and the alternative is apparent. The unworthy members should be reproved. Manufacturers should circulate their trade circulars of course. The benefit is mutual. But let them abstain from publishing the *gratis* half-dozen pages or so of professional matter. The science and art of dentistry should be left to those journals which are exclusively devoted to it, and without being invidious, we would add, to those able to judge of that which should be rejected and of that which should be published. Consequent upon the alternative we have proposed, those taken with *cacoethes scribendi*, not finding such free vent for their delectable efforts, might be induced to bestow time upon their subjects and eventually produce something of value.

The English periodicals are yet *in embryo* and should not be judged harshly; yet strict justice, as well as tender regard, induces us to advise the use of the knife. In England, Americans are noted for using knives, therefore, our present advice will not suggest thoughts of a Franco-American alliance. We mean the surgeon's knife, or more properly the pruning shears, as we look at them from a gardener's point of view. They are the semi-respectable fruits of our toil.

We remark chiefly in respect to the periodical literature of dentistry in England, that the rotten branches of Plagiarism and Disingenuousness should be removed. They are choking the real healthy undergrowth. When English writers desire to borrow material for their work, why let them borrow. We confess, though, that the manner in which our material is re-hashed and published gives us great pleasure. It indicates ability and good taste. We commend paper, type and printing. But these mere externals do not cause us to forget; when we see the meanness of the motives which actuate them we are indignant, but reflecting that they are not yet grown, we sink into regret mixed with contempt, fearing for their unpromising maturity.

As for France and Germany, they are as yet without anything creditable in the way of a periodical devoted to dentistry. The only periodicals we know of are without any especial merit, being composed for the most part of extracts from hospital reports and translations from American publications. This is the more remarkable, as we look to these countries for developments in the microscopic anatomy of the teeth. Still we should remember in this connection, that the recent books on or relating to the teeth were not written by dentists. As examples, we may mention Kölliker and Magitot.

In conclusion then, and as we have already remarked, notwithstanding numerical force, the periodical literature of dentistry cannot be considered as reflecting credit upon dentists. Sincere, earnest and continuous effort is required. The able-minded deserters should again be brought into the ranks to serve out their term of enlistment; the stratagems of trade should be utterly cut of from the approbation of right minded men; young and old all should be encouraged to study the difficult questions connected with their profession, and then, when all this is accomplished, dentists may be able to point to an extensive and valuable mass of periodical literature as their own.

