# Errata

**Published:** 1859-10

**Authors:** 


Our readers will strike out the word *Dental* from the heading of Article V., on page 520.

